# Characterizing the Mechanisms of Nonopsonic Uptake of Cryptococci by Macrophages

**DOI:** 10.4049/jimmunol.1700790

**Published:** 2018-04-11

**Authors:** Jenson Lim, Christopher J. Coates, Paula I. Seoane, Mariam Garelnabi, Leanne M. Taylor-Smith, Pauline Monteith, Camille L. Macleod, Claire J. Escaron, Gordon D. Brown, Rebecca A. Hall, Robin C. May

**Affiliations:** * Biological and Environmental Sciences, University of Stirling, Stirling FK9 4LA, United Kingdom;; † Department of Biosciences, College of Science, Swansea University, Swansea SA2 8PP, Wales, United Kingdom;; ‡ Institute of Microbiology and Infection, School of Biosciences, University of Birmingham, Birmingham B15 2TT, United Kingdom;; § Protein Reference Unit, South West London Pathology, St. George’s University Hospitals NHS Foundation Trust, London SW17 0QT, United Kingdom; and; ¶ Medical Research Council Centre for Medical Mycology Aberdeen Fungal Group, Institute of Medical Sciences, University of Aberdeen, Aberdeen AB25 2ZD, United Kingdom

## Abstract

The pathogenic fungus *Cryptococcus* enters the human host via inhalation into the lung and is able to reside in a niche environment that is serum- (opsonin) limiting. Little is known about the mechanism by which nonopsonic phagocytosis occurs via phagocytes in such situations. Using a combination of soluble inhibitors of phagocytic receptors and macrophages derived from knockout mice and human volunteers, we show that uptake of nonopsonized *Cryptococcus neoformans* and *C. gattii* via the mannose receptor is dependent on macrophage activation by cytokines. However, although uptake of *C. neoformans* is via both dectin-1 and dectin-2, *C. gattii* uptake occurs largely via dectin-1. Interestingly, dectin inhibitors also blocked phagocytosis of unopsonized Cryptococci in wax moth (*Galleria mellonella*) larvae and partially protected the larvae from infection by both fungi, supporting a key role for host phagocytes in augmenting early disease establishment. Finally, we demonstrated that internalization of nonopsonized Cryptococci is not accompanied by the nuclear translocation of NF-κB or its concomitant production of proinflammatory cytokines such as TNF-α. Thus, nonopsonized Cryptococci are recognized by mammalian phagocytes in a manner that minimizes proinflammatory cytokine production and potentially facilitates fungal pathogenesis.

## Introduction

*Cryptococcus neoformans* and *C. gattii* are encapsulated human fungal pathogens that cause cryptococcosis in immunocompromised and, more rarely, immunocompetent individuals. Often found as free-living cells in soil and avian excreta, Cryptococci are not intrinsic human pathogens. However, Cryptococci become human pathogens because several defense mechanisms possessed by these fungi also act as virulence factors within a human or animal host (1), including the ability, firstly, to survive and replicate within free-living soil amoeba and, secondly, to evade clearance by the host immune system by hiding and persisting within macrophages (2, 3).

As Cryptococci enter hosts via inhalation into the lungs, they are detected and phagocytosed by resident alveolar macrophages (4). Phagocytosis is a multistep process that sequentially involves receptor-mediated particle recognition, actin-driven uptake, phagosome maturation, and particle clearance. It is critical during the early innate immune response to ensure the removal of microorganisms and apoptotic cells as well as subsequent priming of the adaptive immune response through the production and release of cytokines, such as TNF-α (5). Phagocytosis of Cryptococci is typically inefficient unless they are opsonized (coated) by Abs or complement proteins found in serum within the circulatory system. Interestingly, there is a lack of serum opsonins in the alveoli of the lungs, and so the initial uptake of *Cryptococcus* upon colonization is most likely through a nonopsonized route (6).

Nonopsonic phagocytosis requires host cell phagocytic pattern recognition receptors (PRRs) to directly recognize fungal cell wall components (pathogen-associated molecular patterns [PAMPs]) (7) such as β-glucans or mannan polysaccharides, but the nature of this interaction for Cryptococci remains unknown. In this article, we show that nonopsonized *C. neoformans* and *C. gattii* enter macrophages in a spleen tyrosine kinase (Syk)–dependent, mannose receptor–independent manner that involves the receptors Dectin-1 and Dectin-2. This differential uptake of *C. neoformans* and *C. gattii* corresponds to differential exposure of PAMPs found on the fungal cell wall. Phagocytic kinetics of macrophages and insect hemocytes in the absence or presence of cellular receptor inhibitors were similar in response to fungal targets. Finally, we demonstrate that entry of *Cryptococcus* does not affect NF-κB nuclear translocation or subsequent TNF-α release, highlighting the remarkably noninflammatory capabilities of this organism.

## Materials and Methods

### Reagents

All reagents (e.g., heat-inactivated FBS, DMEM, l-glutamine, powdered yeast-extract peptone dextrose, and PBS) were purchased from Sigma-Aldrich unless stated otherwise. Mouse macrophage–CSF (130-094-129) and human GM-CSF (130-093-862) were purchased from Miltenyi Biotec. Commercially sourced inhibitors tested included the Syk–inhibiting plant metabolite, Piceatannol (527948; Calbiochem); the β-1,3-glucan from brown algae *Laminaria digitata*, Laminarin (L9634; Sigma-Aldrich); and mannan from *Saccharomyces cerevisiae* (M7504; Sigma-Aldrich).

The Abs used in this study were rabbit anti-sheep RBCs, IgG fraction (#55806; MP Biomedicals); rabbit anti-sheep RBCs, IgM fraction (CL9000M; VH Bio/Cedarlane); rabbit anti–65 kDa subunit (p65) NF-κB mAb (clone D14E12, #8242; New England Biolabs/Cell Signaling Technology); rat anti-αM (clone 5c6, MCA2289; Bio-Rad AbD Serotec); and rabbit anti–Phospho-Syk (Tyr525/526 in humans, Tyr519/520 in mice, clone C87C1, #2710; New England Biolabs/Cell Signaling Technology), a kind gift from Y. Senis (University of Birmingham). Rhodamine-Phalloidin and Alexa Fluor–conjugated secondary Abs were purchased from Life Technologies and Calcofluor White from Sigma-Aldrich. Glucan-6-phosphate and mouse anti-cryptococcal capsule Ab (clone 18B7) were kind gifts from D. Williams (East Tennessee State University) and A. Casadevall (Albert Einstein College of Medicine), respectively.

### Mice

Mice devoid of specific PRRs (in C57BL/6 background) were reported previously (8, 9) and were housed under pathogen-free conditions in the registered animal facility at the University of Aberdeen. Mice were allocated to experimental groups on the basis of genotype and age-matching. All animal procedures were performed according to the protocols provided by the Animal Welfare and Ethical Review Body of the University of Aberdeen and are regulated by the UK Home Office Animal (Scientific Procedures) Act of 1986 and European Directive 2010/63/EU.

### Yeast and bacterial cell growth conditions

*C. neoformans* strain H99, *C. gattii* strain R265, and *Candida albicans* strain SC5314 were incubated in liquid yeast-extract peptone dextrose medium for 24 h (unless stated otherwise) at 25°C on a rotator at 20 rpm (or 37°C, 200 rpm for *C. albicans*). *Escherichia coli* strain DH5α was incubated in Luria-Bertani broth for 16 h at 37°C in a shaking incubator at 200 rpm. Yeast cells were centrifuged at 3000 × *g* for 2.5 min (or 6000 × *g* for 1 min for *E. coli*), washed three times in PBS, and counted with a hemocytometer prior to use.

### Mammalian cell growth conditions

Cells from the murine macrophage–like cell line J774.A1 (American Type Culture Collection number TIB-67) were cultured in DMEM supplemented with 2 mM l-glutamine and 10% heat-inactivated FBS at 37°C, 5% CO_2_ (10). As required, macrophages were scrapped in PBS, counted, and seeded (50,000/well) onto 13 mm acid-washed glass coverslips, and incubated for 24 h at 37°C, 5% CO_2_ prior to experimental use.

Macrophages devoid of specific PRRs were derived from mouse bone marrow. Bone marrows were flushed using a 21-gauge needle from the hind leg bones of either receptor knockout (KO) or litter-matched wild type (WT) mice. Monocytes were differentiated into macrophages with 20 ng/ml M-CSF (Miltenyi Biotec) for 7 d.

Pooled PBMCs were isolated from whole blood from healthy volunteers using density gradient centrifugation with Ficoll-Paque (GE Healthcare). The mononuclear layer was collected and washed with PBS to remove platelets. Monocytes were purified by adherence to plastic in RPMI 1640 media supplemented with 5% heat-inactivated FBS, 2 mM glutamine, 100 mg/ml streptomycin, and 100 U/ml penicillin at 37°C, 5% CO_2_ for 1 h. Nonadherent cells were removed with PBS and adherent cells differentiated into macrophages with 20 ng/ml recombinant human GM-CSF (Miltenyi Biotec) for 7 d. This study was covered by the University of Birmingham’s Science, Technology, Engineering, and Mathematics Ethical Review Committee.

### Phagocytic challenge

Macrophages were serum starved for 2–16 h with serum-free medium at 37°C, 5% CO_2_. Where needed, inhibitors were added directly and left for a further 30 min. Next, media were removed prior to fresh serum-free medium being added containing either 1 μg/ml 18B7 Ab (against cryptococcal capsule) or unopsonized targets at a multiplicity of infection of either 10:1 or 20:1 for 20–180 min at 37°C, 5% CO_2_. Cells were washed three times with PBS to remove unbound yeast/bacteria cells and fixed in 4% paraformaldehyde for 10 min at room temperature (RT).

### Galleria mellonella maintenance

Larvae of the greater wax moth, *Galleria mellonella*, were sourced from Livefoods Direct (U.K.) and stored in wood shavings in the dark at 13°C. This study was covered by the University of Stirling’s Animal Welfare and Ethical Review Body. Healthy larvae weighing between 0.2 and 0.4 g were used in all experiments. Larvae were inoculated with different concentrations of inhibitors via intrahemocele injection 1 h prior to infection with 1 million *C. neoformans* H99 per larva as described previously (11). Controls consisted of larvae that received a 20 μl PBS inoculum. Three to five larvae were used per treatment, with all treatments being performed on at least three independent occasions.

For phagocytosis, larvae were bled and hemolymph treated as previously described (12). Briefly, pooled hemolymph was mixed with 0.5 ml PBS and added onto a 13 mm coverslip in a 24-well plate. Hemocytes were centrifuged onto the coverslips for 10 min at 500 × *g* at RT before washing three times with PBS to remove noninternalized yeasts. Cells were then fixed with 4% paraformaldehyde before permeabilization and immunostained as described below. All determinations were performed on at least three independent occasions.

### Immunofluorescence and scoring

Fixed cells on coverslips were permeabilized with 0.1% Triton X-100 for 5 min (if necessary to identify internalized yeasts), washed with PBS, and blocked with 0.5% BSA in PBS for 30 min. Appropriate primary Abs (1:200 dilution) were added to cells, left for 30 min at RT, washed with PBS, and counterstained with the appropriate fluorophore–conjugated secondary Ab, along with Rhodamine-Phalloidin and Calcofluor White. Coverslips were then washed in PBS and distilled water before mounted in ProLong Gold Antifade Reagent (Life Technologies) and analyzed by microscopy.

For counting of phagocytosed yeast/bacteria, fixed but unpermeabilized cells on coverslips were stained with Calcofluor White to highlight the external yeasts. Coverslips were analyzed with a Nikon Eclipse Ti microscope under a 63× oil immersion objective. Between 5 and 10 fields of view of each coverslip were counted for number of macrophages and association of microbial cells. At least 100 macrophages were observed for each coverslip.

The enrichment in phosphorylated Syk at sites of yeast binding and the translocation of p65 into the nucleus during NF-κB activation were studied and scored by the Nikon A1R confocal microscope using 20× to 63× objectives. For the former, a minimum of 25 infected cells per condition were analyzed for a discrete local enrichment in marker signal (Syk) at bound particles. For the latter, between three and five fields of view for each sample/coverslip were counted for the number of macrophages with p65 marker signal located within the nucleus and expressed as a percentage of the total number of macrophages (%NF-κB nuclear translocation).

### In vitro cytokine production

J774.A1 and primary human macrophages were cultured in 96-well microtiter plates (Greiner) at 10,000 cells/well in a final volume of 200 μl. Cells were stimulated with either control medium, LPS, or a range of unopsonized pathogenic yeasts. After 6 h of incubation at 37°C, plates were centrifuged (500 × *g* for 10 min), and the supernatant was collected and stored at −80°C until cytokine assays were performed. Levels of TNF-α were determined by commercial ELISA kits, used according to the instructions of the manufacturer (R&D Systems).

### Statistical analyses

Analysis carried out on the results described in this paper was by a generalized linear model using a Poisson error distribution in R (R Development Core Team). This was tested for significance using a post hoc Tukey honest significant difference (HSD) test.

## Results

### Uptake of nonopsonized Cryptococci via mannose receptor is activation dependent

**FIGURE 1.**
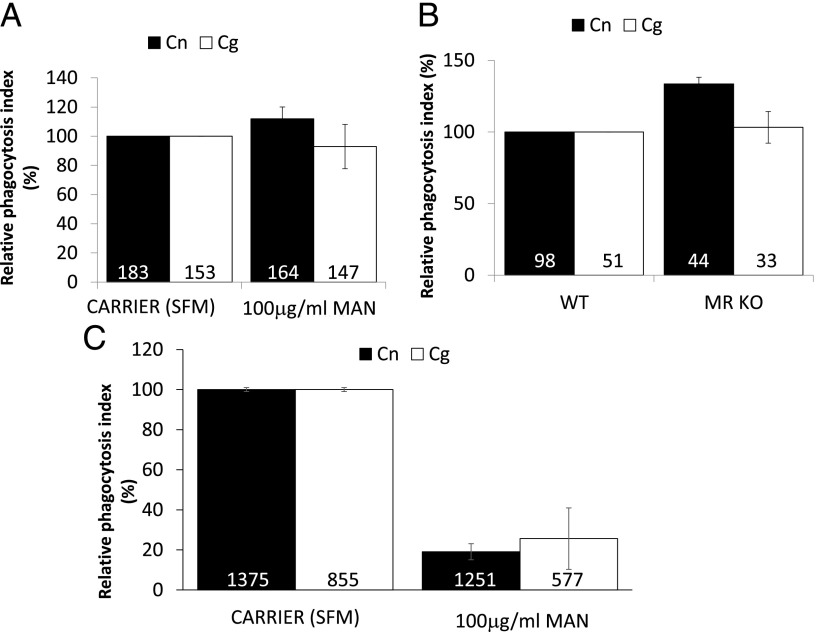
Mannose receptor is important but dispensable during uptake of *Cryptococcus* particles. Mouse macrophage cell line J774.A1 (**A**) or differentiated BMMs (**B**) (WT or MR KO) or differentiated primary human macrophages (**C**) were challenged with either *C. neoformans* H99 (Cn, black bars) or *C. gattii* R265 (Cg, white bars) for 60 min, processed for immunofluorescence, and scored for phagocytosis as described in *Materials and Methods*. Where indicated, J774.A1 and primary human macrophages were pretreated with 100 μg/ml mannan (MAN) for 30 min before the addition of *Cryptococcus* particles. Phagocytosis indices were related to the values obtained from the negative controls. Number in bars indicate the total number of phagocytes counted. Results are expressed as the mean ± SD of at least three independent experiments.

**FIGURE 2.**
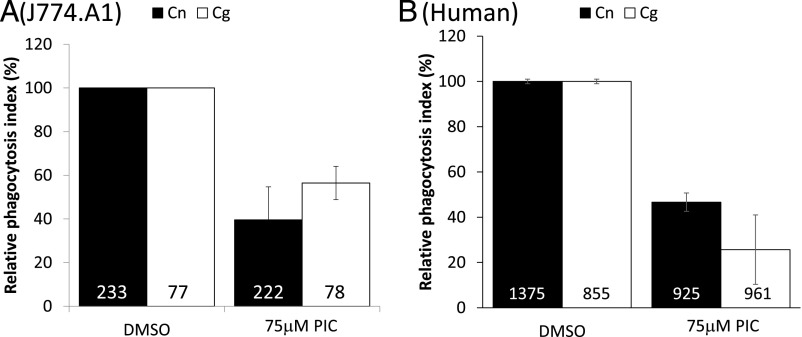
Uptake of *Cryptococcus* particles is Syk-dependent. Mouse macrophage cell line J774.A1 (**A**) or differentiated primary human macrophages (**B**) were challenged with unopsonized *C. neoformans* H99 (Cn, black bars) or *C. gattii* R265 (Cg, white bars) for 60 min, processed for immunofluorescence, and scored for phagocytosis as described in *Materials and Methods*. Phagocytosis indices were related to the values obtained from the negative controls. Number in bars indicate the total number of phagocytes counted. Results are expressed as the mean ± SD of at least three independent experiments.

As previously observed, the levels of nonopsonic uptake of Cryptococci is very low (e.g., 0.4% of *C. neoformans* serotype D was taken up by unstimulated mouse peritoneal macrophages; or 7–21% of *C. gattii* R265 was taken up by human dendritic cells (DCs); (13, 14)) and our results are in agreement with those findings – 8.89 or 5.83% of primary human macrophages contained one or more *C. neoformans* H99 or *C. gattii* R265, respectively (based on the carrier controls in Figs. 1C, 2B), after 2 h of incubation. The mannose receptor is broadly expressed on macrophages and important for the nonopsonic uptake of fungal pathogens such as *C. albicans* and *Pneumocystis carinii* (15, 16). The uptake of *C. neoformans* H99 or *C. gattii* R265 by J774.A1 macrophages pretreated with soluble mannan (a competitive inhibitor of mannose receptor binding) was unaltered relative to control (untreated) cells (Fig. 1A). Similarly, M-CSF differentiated bone marrow macrophages (BMMs) from mannose receptor KO mice (MR KO) showed no reduction in uptake of either *C. neoformans* or *C. gattii* relative to WT control cells (Fig. 1B). Interestingly, however, GM-CSF–differentiated primary human macrophages showed a strong inhibition of uptake under the same conditions (Fig. 1C), suggesting that the mannose receptor may play a greater role in cryptococcal uptake into human cells than those of mice.

### Phagocytosis of unopsonized Cryptococci is Syk-dependent

**FIGURE 3.**
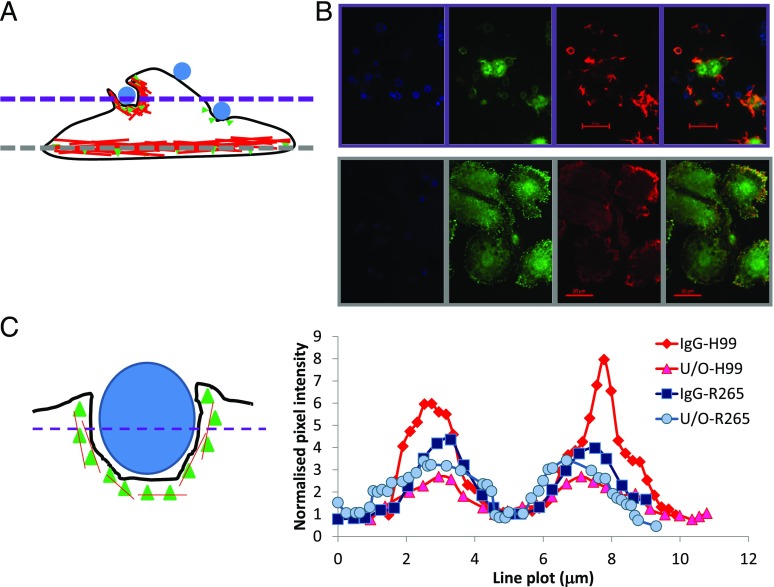
Activated Syk is essential for the uptake of *Cryptococcus* particles. Mouse macrophage cell line J774.A1 was challenged with either (IgG-opsonized or unopsonized, U/O) *C. neoformans* H99 or *C. gattii* R265 for 15 min (**B**), processed for immunofluorescence, and analyzed by confocal microscopy of localized phospho-Syk (B and **C**) as described in *Materials and Methods*. (**A**) Schematic diagram J774.A1 macrophage with intracellular actin cytoskeleton (red) and yeast particles (blue). To confirm phospho-Syk localization, the bottom of the cells was observed first [(A), grey dashed line and (B), bottom panels], before moving to the middle of the cells [(A), purple dashed line, (B), top panels]. Pixel intensities for 20 cells per sample were determined [(C), right] and normalized to the intensity at the center of the cell [(C), left]. (A and C) The green triangles denote phospho-Syk. The black line denotes the outline of a cell as imagined from the side (i.e., its *z*-axis). Results are expressed as the mean ± SD of at least three independent experiments. Scale bar, 20 μm.

The other major class of nonopsonic phagocytic receptors for fungi are the dectins (17). Both dectin-1 and dectin-2 require Syk activity for their function, via ITAMs contained within dectin-1 itself or via membrane association with ITAM–containing Fc receptor γ-chain in the case of dectin-2 (18). Inhibiting Syk activity in J774.A1 cells by using piceatannol (19) resulted in a marked reduction in their ability to phagocytose either *C. neoformans* H99 or *C. gattii* R265 (Fig. 2A, *p* < 0.05). The same observation was also seen in GM-CSF–differentiated primary human macrophages from pooled monocytes isolated from human volunteers (Fig. 2B). In line with this, staining with an anti–Phospho-Syk Ab showed intense accumulation of active Syk at phagocytic cups forming around nonopsonized Cryptococci (Fig. 3). This Ab was raised against the tyrosine phosphorylated residues at positions 525 and 526, located in the activation loop of the Syk kinase domain and essential for Syk function (20). Therefore, we propose that the localization of this Ab to the sites of nonopsonic uptake of Cryptococci and the activity of piceatannol in blocking uptake suggests that Syk activity is required for internalization.

### Phagocytosis of unopsonized Cryptococci is partially dependent on Dectin-1

**FIGURE 4.**
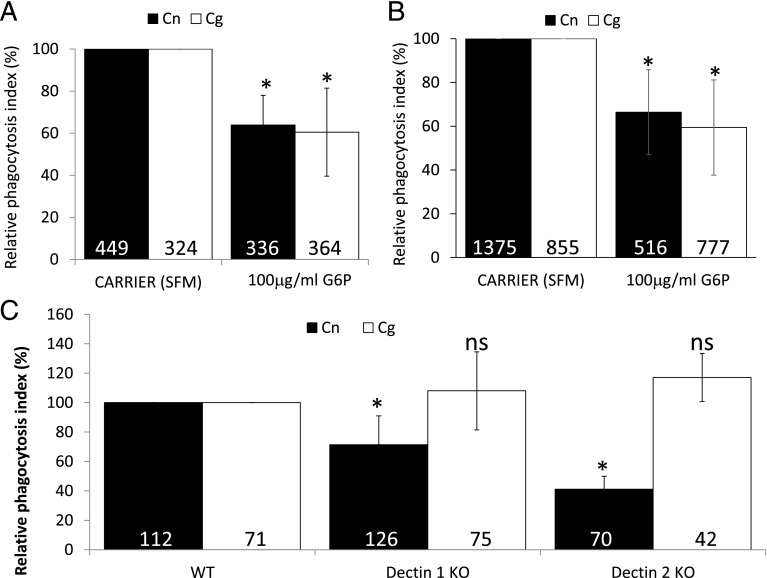
Dectins are required for uptake of *Cryptococcus* particles. Mouse macrophage cell line J774.A1 (**A**), differentiated primary human macrophages (**B**), or differentiated BMMs (**C**) (WT, Dectin-1 KO, or Dectin-2 KO) were challenged with either *C. neoformans* H99 (Cn, black bars) or *C. gattii* R265 (Cg, white bars) for 60 min, processed for immunofluorescence, and scored for phagocytosis as described in *Materials and Methods*. Where indicated, J774.A1 were pretreated with 100 μg/ml glucan-6-phosphate (G6P) for 30 min before the addition of *Cryptococcus* particles. Phagocytosis indices were related to the values obtained from the negative controls. Number in bars indicate the total number of phagocytes counted. Results are expressed as the mean ± SD of at least three independent experiments. *p < 0.05. ns, not significant (*p* ≥ 0.05).

To test for a role for the dectin family of receptors during phagocytic uptake of nonopsonized *Cryptococci*, we first exposed J774.A1 macrophages (Fig. 4A) or differentiated primary human macrophages (Fig. 4B) to the dectin-1 inhibitor glucan-6-phosphate before challenging with either unopsonized *C. gattii* R265 or *C. neoformans* H99. This inhibitor partially blocked the uptake of both species of *Cryptococcus*, suggesting dectin-1 contributes toward Cryptococci uptake but is not the sole recognition receptor involved in this process (Fig. 4A, 4B). In line with this, M-CSF–differentiated BMMs from dectin-1 and dectin-2 KO mice both showed substantially impaired uptake of *C. neoformans* H99 – surprisingly, this was not the case for *C. gattii* R265 (Fig. 4C). This suggests either the presence of another Syk-dependent nonopsonic receptor or that both dectins are redundant with each other for *C. gattii*, but not *C. neoformans* uptake.

### Nonopsonic uptake in the Galleria model

**FIGURE 5.**
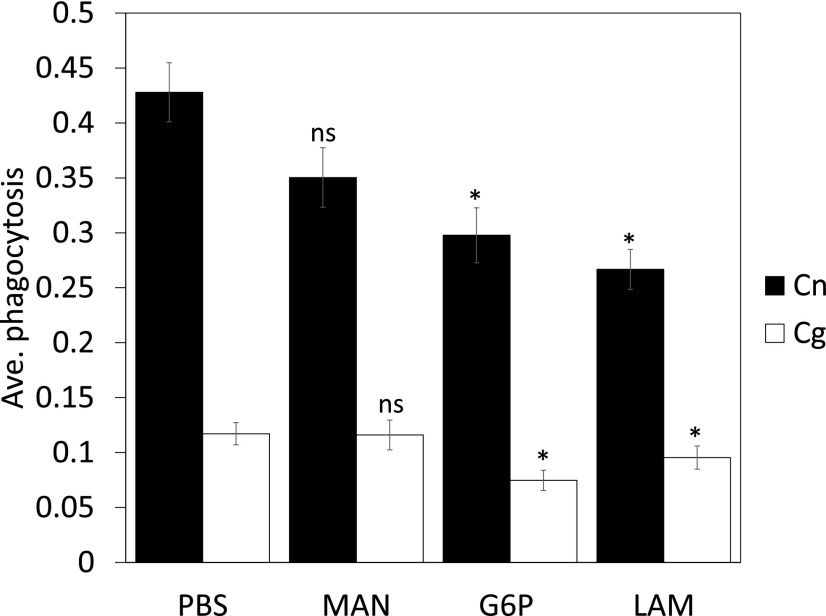
Administration of polysaccharides blocks uptake of *Cryptococcus* particles to hemocytes in the *G. mellonella* larvae model. Larvae were inoculated with 60 μg of blocking sugars 1 h prior to infection for 2 h with 10^6^ *C. neoformans* H99 (black bars) or *C. gattii* R265 (white bars). Uptake of yeast of hemocytes was determined under light microscopy. Results are expressed as the mean ± SD of at least three independent experiments. *p < 0.05 (related to PBS control). ns, not significant (*p* ≥ 0.05).

The greater wax moth, *G. mellonella*, is widely used as a model organism in the study of host–pathogen interactions with a variety of human pathogens (21). As with other insects, *G. mellonella* does not possess an adaptive immune system like mammals but possesses a complex innate immune system that includes phagocytic cells, termed hemocytes (22, 23). We therefore tested whether nonopsonic uptake of Cryptococci in *G. mellonella* showed similar receptor dependency as in mammalian cells by pretreating larvae for 1 h with soluble mannan, glucan-6-phosphate, or laminarin. The full genome sequence of *Galleria* is currently available but not fully annotated (24). However, several β-1,3-glucan–binding protein analogs and C-type lectins have been characterized in this species, as well as other Lepidopterans, namely *Manduca sexta* (25, 26), *Bombyx mori* (27), and *Plodia interpunctella* (28). Recognition of fungal PAMPs (e.g., curdlan and mannan) by membrane-bound receptors modulate cellular (hemocyte)-directed immunity in insects (encapsulation, nodulation, and phagocytosis) (29). Although soluble mannan did not significantly reduce association of Cryptococci with *Galleria* hemocytes in data presented in this paper, both glucan-6-phosphate and laminarin led to a marked reduction in uptake (Fig. 5, *p* < 0.001 for both when compared to the PBS controls).

**FIGURE 6.**
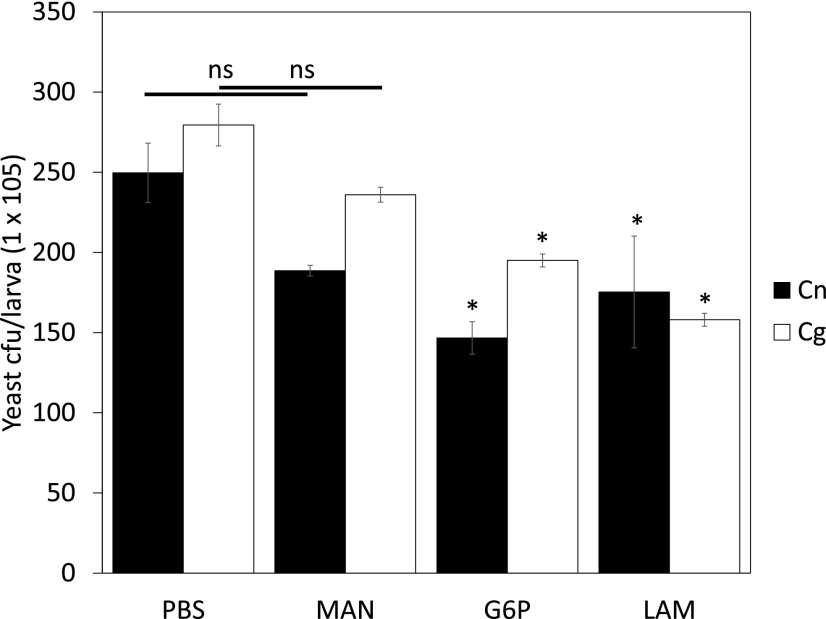
Glucan administration protects *G. mellonella* larvae from infection by *C. neoformans* or *C. gattii.* Larvae were inoculated with 60 μg of blocking sugars 24 h prior to infection for a further 24 h with 10^6^ *C. neoformans* H99 (black bars) or *C. gattii* R265 (white bars). Fungal load was determined by serially diluting homogenized larvae and plating aliquots onto erythromycin containing agar plates. Yeast cell density were related to the values obtained from the negative (PBS) controls and expressed as cfu × 10^5^/larva. Results are expressed as the mean ± SD of at least three independent experiments. *p < 0.05 (related to PBS control). ns, not significant (*p* ≥ 0.05).

Interestingly, administering glucan-6-phosphate or laminarin for 24 h appeared to partially protect the insect larvae from infection by both unopsonized species of *Cryptococcus* (Fig. 6), suggesting that disease establishment in this model organism requires the fungus to grow intracellularly, something that has previously been proposed for human hosts (30).

### Cryptococcal uptake by macrophages does not lead to increased proinflammatory cytokine secretion

**FIGURE 7.**
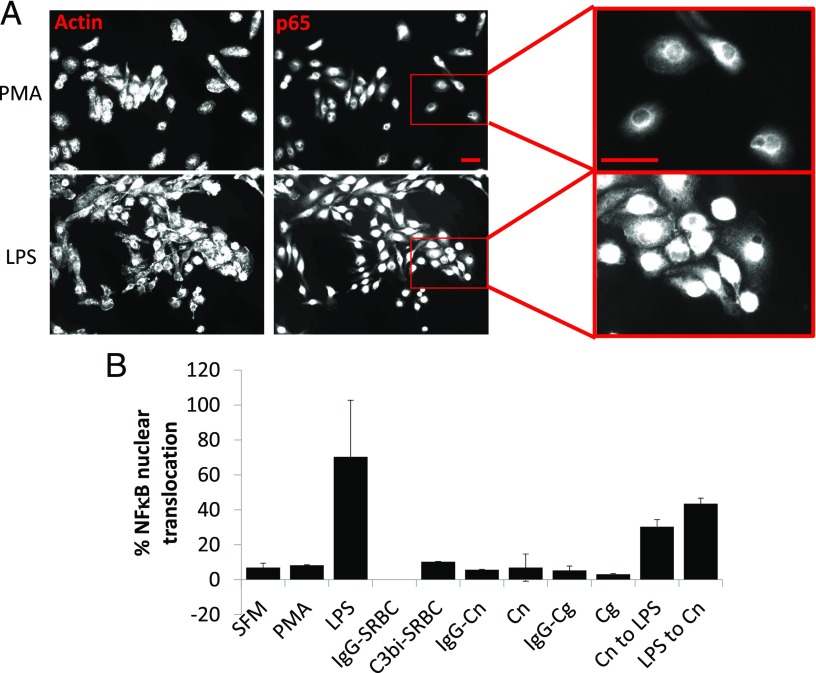
Uptake of *Cryptococcus* did not affect NF-κB nuclear translocation. J774.A1 macrophages were challenged with a variety of opsonized or unopsonized pathogenic fungi, SRBCs, or soluble agonists (LPS or PMA), processed for immunofluorescences, analyzed by microscopy (**A**), and scored for p65 nuclear translocation (**B**), as described in *Materials and Methods*. (A) Representative images of PMA- (top) or LPS- (bottom) stimulated J774.A1 macrophages and stained to highlight either actin or p65. Actin was stained using rhodamine-phalloidin; p65 was stained using the anti–65 kDa subunit (p65) NFκB mAb with an anti-rabbit Alexa Fluor–488. Scale bar, 20 μm.

Unlike many pathogens, internalization of opsonized Cryptococci into phagocytes is not accompanied by the production of proinflammatory cytokines such as TNF and IL-1α or IL-1β (31, 32). To test whether this is also true of nonopsonic uptake, we measured the secretion of TNF-α and nuclear translocation of p65 (a major regulator of cytokine transcription) from J774.A1 macrophages upon challenge with unopsonized or serum-opsonized *C. neoformans* H99 or *C. gattii* R265. Although LPS-stimulated macrophages showed strong nuclear translocation of p65, neither IgG-opsonized nor unopsonized *C. neoformans* H99 or *C. gattii* R265 stimulated NF-κB activation (Fig. 7A). However, NF-κB activation could be restored in cryptococcal exposed macrophages by the subsequent addition of LPS (Fig. 7B).

**FIGURE 8.**
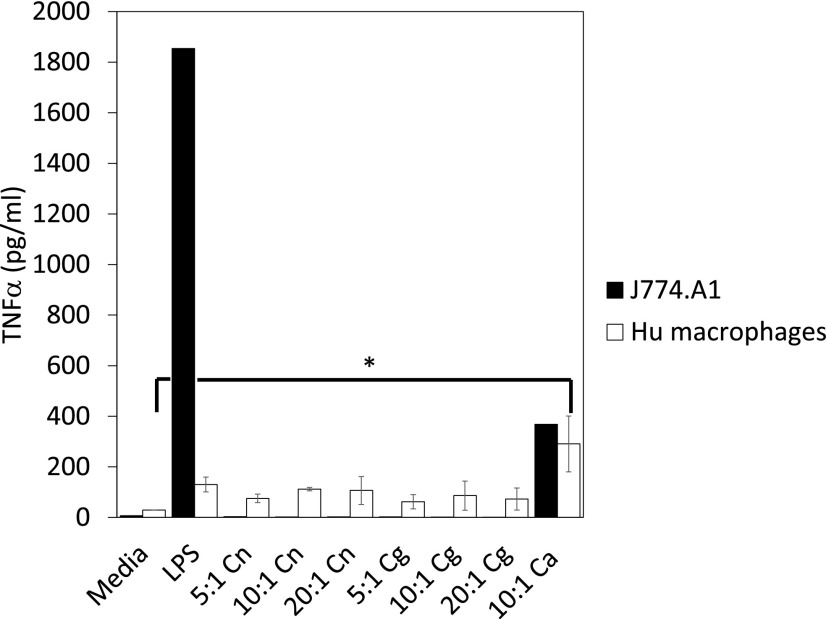
Uptake of *Cryptococcus* did not affect proinflammatory cytokine response. J774.A1 macrophages (black bars) or differentiated primary human macrophages (white bars) were challenged with a variety of unopsonized pathogenic fungi (*C. neoformans*, Cn; *C. gattii*. Cg; *Candida albicans*, Ca) or LPS, and subsequent supernatants were analyzed by ELISA, as described in *Materials and Methods*. Results are expressed as the mean ± SD of at least three independent experiments. *p < 0.05.

Furthermore, to test whether internalization of unopsonized Cryptococci into J774.A1 mouse macrophages or primary human macrophages elicits the production of proinflammatory cytokines such as TNF, we measured the secretion of TNF-α from J774.A1 macrophages or primary human macrophages upon challenge with unopsonized *C. neoformans* H99 or *C. gattii* R265, with *C. albicans* and LPS as controls. With J774.A1 mouse macrophage and primary human macrophages, *C. albicans*- or LPS-stimulated macrophages showed stronger TNF-α production compared to varying doses of *C. neoformans* H99 or *C. gattii* R265 (Fig. 8; *p* = 0.04 for *C. albicans* versus media control, *p* > 0.05 for *C. albicans* versus *C. neoformans*/*C. gattii*). Overall, this suggests that Cryptococci do not actively block inflammatory signaling in host cells and do not induce a strong inflammatory stimulus following nonopsonic uptake.

## Discussion

In this study, we examined the phagocytic uptake of unopsonized cryptococcal yeast particles by macrophages. This process relies on the use of phagocytic receptors, which can be categorized either as opsonic or nonopsonic. Opsonic phagocytic receptors include the Fc receptor and complement receptor families, which recognize Ab- or complement-opsonized (coated) particles, respectively. Nonopsonic phagocytic receptors are PRRs, such as the C-type lectin family of receptors, which recognize distinct PAMPs on the fungal surface (33).

Although phagocytosis of *Cryptococcus* within the circulatory system would occur predominantly through an opsonized (coated) uptake route because of the presence of Abs and/or complement proteins found in serum, this is not always the case. For example, the first encounter of the human body with *Cryptococcus* is through the lungs when desiccated yeast cells or spores are breathed in. These cryptococcal particles encounter their initial immunological challenge through resident alveolar macrophages and DCs in a serum-deficient or low-serum environment (34–36). Interestingly, it was reported recently that between 25 and 40% of mouse lung-resident macrophages are able to phagocytose *C. neoformans* particles through a scavenger receptor pathway (37). Therefore, this confirms that initial uptake of *Cryptococcus* by macrophages is most likely through a nonopsonized route, and there is a need to understand the mechanisms that underpin this process (6). We confirmed that, compared with the bacterium *E. coli* or fungus *C. albicans*, Cryptococci cells are not readily taken up by mammalian macrophages, most likely because of the presence of the capsule, which renders Cryptococci antiphagocytic (38, 39). By using a combination of a soluble mannose inhibitor and MR KO tissue, we demonstrated that mannose receptor was not necessary for the uptake of either species of *Cryptococcus*, in line with recent data from the zebrafish model (40), although this is not the case in primary human macrophages. We note that others have shown MR KO mice to be more susceptible to *C. neoformans* (41) and demonstrated a role for this receptor, along with FcγRII (CD32) in driving cryptococcal uptake into DCs (42). Thus, mannose receptor dependency apparently varies across different cell types and tissue contexts.

Next, we pursued a different set of nonopsonic PRRs, dectin-1 and dectin-2, which are C-type lectin receptors that are highly expressed in macrophages and are key β-glucan receptors (43, 44). Recognition of soluble or surface expressed β-glucans on yeasts is sufficient to initiate and mediate phagocytosis and proinflammatory cytokine responses (45). Both of these receptors require Syk activity (18, 46, 47), and, indeed, our data clearly demonstrate the activation of Syk at phagocytic cups containing unopsonized Cryptococci, as well as a strong dependency on Syk for particle uptake. Interestingly, pharmacological inhibition of dectins inhibited uptake of both *C. neoformans* and *C. gattii* in J774.A1 mouse and human macrophages, but BMMs from dectin-1– and dectin-2–KO mice showed defects only in the uptake of *C. neoformans* and not *C. gattii*, an effect that has been observed before (48). The most parsimonious explanation is therefore that the two dectin receptors are redundant for the uptake of *C. gattii*, but not *C. neoformans*, perhaps reflecting differing surface components between the two species, as reported recently (49). Such surface variation between species, strains, and potentially developmental stages of Cryptococci may explain many of the previous inconsistencies in the literature regarding dectin dependency (or otherwise) (50, 51).

Alongside mouse macrophages, we adopted wax worm larvae (*G. mellonella*) as an alternative model for understanding cryptococcal virulence and host immune responses (52–54) in which cryptococcal phagocytosis has previously been reported (55). Our data demonstrate striking similarities in patterns of uptake between this invertebrate host and murine phagocytes. In addition, we showed that inhibiting phagocytosis in this alternative host reduces disease burden, highlighting the importance of host phagocytes as a niche for cryptococcal replication.

We acknowledge that although there are currently no direct dectin receptor homologs identified in *G. mellonella*, many C-type lectins have been characterized in other insect models, for example; the tobacco hornworm, *Manduca sexta* (immulectin-2 facilitates phagocytosis of bacteria (56)); webworm, *Hyphantria cunea* (lectin (57, 58)); silkworm, *Bombyx mori* (BmLBP and BmMBP (59, 60)); and the cockroach, *Blaberus discoidalis* (a β-glucan–specific lectin (61)). These invertebrate C-type lectins show up to 35% similarity with mammalian C-type lectins and can bind to several PAMPs, including LPS, lipoteichoic acid, and β-glucan and are inducible when the host is exposed to microbial challenge or ligands and the mechanisms for uptake of pathogenic microbes by *G. mellonella* hemocytes are similar to that of human neutrophils (62).

Two key reports have shown that there are at least three scavenger receptors involved in the recognition of different serotypes of *Cryptococcus neoformans*, namely the homologous genes from the nematode *Caenorhabditis elegans*, CED-1 and C03F11.3, as well as the mouse MARCO scavenger receptors (37, 63). Interestingly, knocking out MARCO gene from mice did not abolish uptake of *C. neoformans* by lung-resident mononuclear phagocytes (37), suggesting a role or roles for the extent and distribution of multiple receptors and ligands on the surface of both host cell and yeast.

Finally, we demonstrate that entry of *Cryptococcus* does not affect NF-κB nuclear translocation and its subsequent TNF-α release in the Dectin-1/Syk/NF-κB signaling axis—both in J774.A1 mouse macrophages and in primary human macrophages. Although it is known that Dectin-1 coupling to Syk leads to downstream activation of NF-κB, which coordinates the transcription of innate response genes, including expression of proinflammatory cytokines such as TNF-α (64–66), this appears not to be the case for cryptococcal uptake.

In conclusion, we propose that unopsonized Cryptococci are recognized and engulfed via mannose receptor- or dectin-based recognition in vitro depending on the activation state of the host cells. The absence of an associated proinflammatory cascade allows the yeast to exploit this intracellular niche for rapid disease establishment.
